# Management of an Unusual Iliac Fossa Venous Plexus

**DOI:** 10.1155/2011/140389

**Published:** 2012-01-09

**Authors:** Irwin M. Best

**Affiliations:** Interventional Radiology, Department of Radiology, Emory University School of Medicine, 1364 Clifton Road, NE, Atlanta, GA 30322, USA

## Abstract

Symptomatic iliac fossa and suprapubic varicosities are uncommon presentations in adults. Such presentations often point to acquired obstructive process to pelvic outflow or to the progression of venous insufficiency and reflux in the pelvic and gonadal veins. Less frequently, venous anomalies of the renal veins or IVC might be implicated. Furthermore, late presentations of congenital or acquired developmental abnormalities might become manifest. As this case illustrates, a thorough understanding of the underlying pathologic process and the anatomical derangement must be sought before any treatment is instituted. Unnecessary extirpation of these varicosities would simply have removed vital physiologic cross-pelvic collateral circulation from the lower extremity in the face of chronic iliac vein occlusion.

## 1. Introduction

Large suprapubic varicosities are rare. In adulthood, an acquired condition from venous stenosis or occlusion should be considered. In younger patients, a congenital or acquired developmental anomaly should be suspected. A thorough initial clinical evaluation is helpful in planning diagnostic and therapeutic interventions as illustrated. 

## 2. Materials and Methods

**Figure 1:**
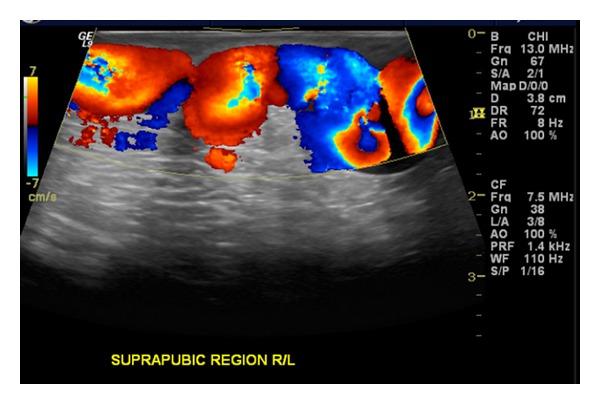
Duplex ultrasound of suprapubic venous plexus. Note the tortuosity in the vessel and the continuous flow beneath the ultrasound probe.

**Figure 2:**
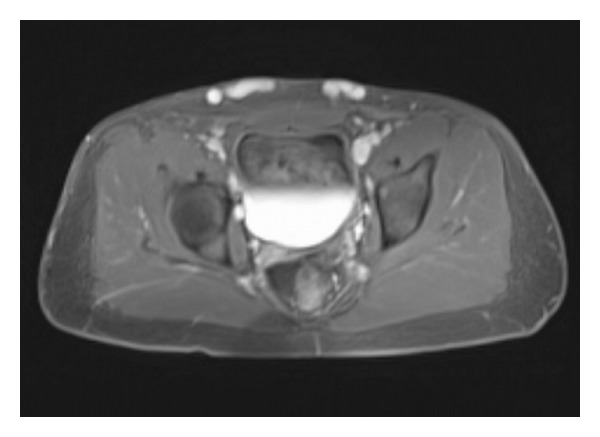
Axial MRI of pelvis with contrast showing large suprapubic varicosities (white arrows) and a small right external iliac (short arrows). The left external iliac vein is larger (long arrows).

**Figure 3:**
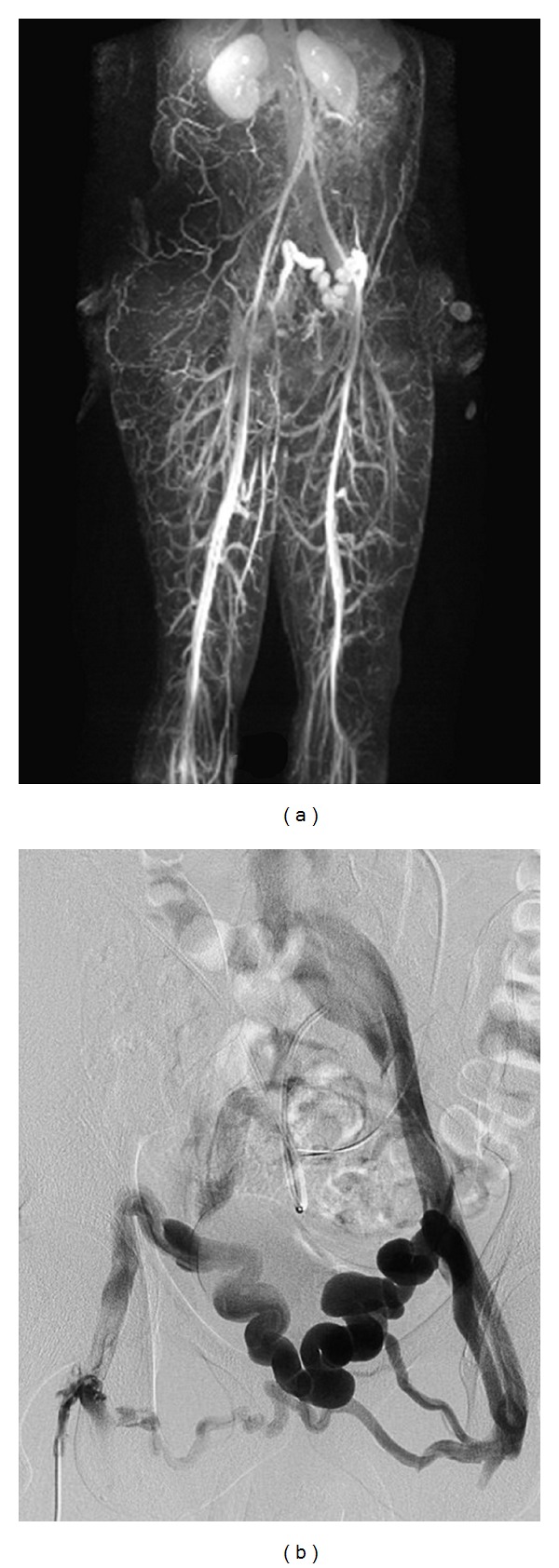
MRI with contrast demonstrating abnormal iliac fossa varicosities (a) Conventional right femoral venogram demonstrating large right to left cross pelvic collaterals to the left femoral system (b).

A 21-year-old presented with a two-month complaint of pain in both iliac fossae as well as suprapubic tenderness particularly after vigorous lower extremity exercise. His medical history was significant for prematurity at 26 weeks. He had a long postnatal ICU course and several intracranial shunts for hydrocephalus. Physical exam revealed large, mildly tender, ballotable suprapubic varicosities extending across the pubis from the right to the left iliac fossa. No Bruit was present over the varices. Both legs were equal in length and there were no other skin abnormalities. Tenderness was also noted along the medial thighs and perineum and over the iliac fossae along the course of the enlarged venous plexus. No palpable chords or intraluminal masses were detected. His color flow duplex scan was negative for deep venous thrombosis. However, Doppler waveforms show continuous forward flow in the large subcutaneous venous plexus, Figure 1. Magnetic venous imaging was obtained to evaluate pelvic and intra-abdominal varices and to better characterize the feeding and outflow vessels.  Figure 2 demonstrated large subcutaneous varicosities and a diminutive right external iliac vein compared to the left iliac vein. The reformatted MRI with Gadolinium, Figure 3(a), demonstrated the subcutaneous plexus and the collateral flow from right femoral system to the left femoral system via these enlarged cross-pelvic collaterals. The subsequent diagnostic conventional venogram is seen in Figure 3(b). The right iliac vein is not identified in these series, and venous flow is from the right groin to the left via cross-pelvic collateral channels.

## 3. Results

**Figure 4:**
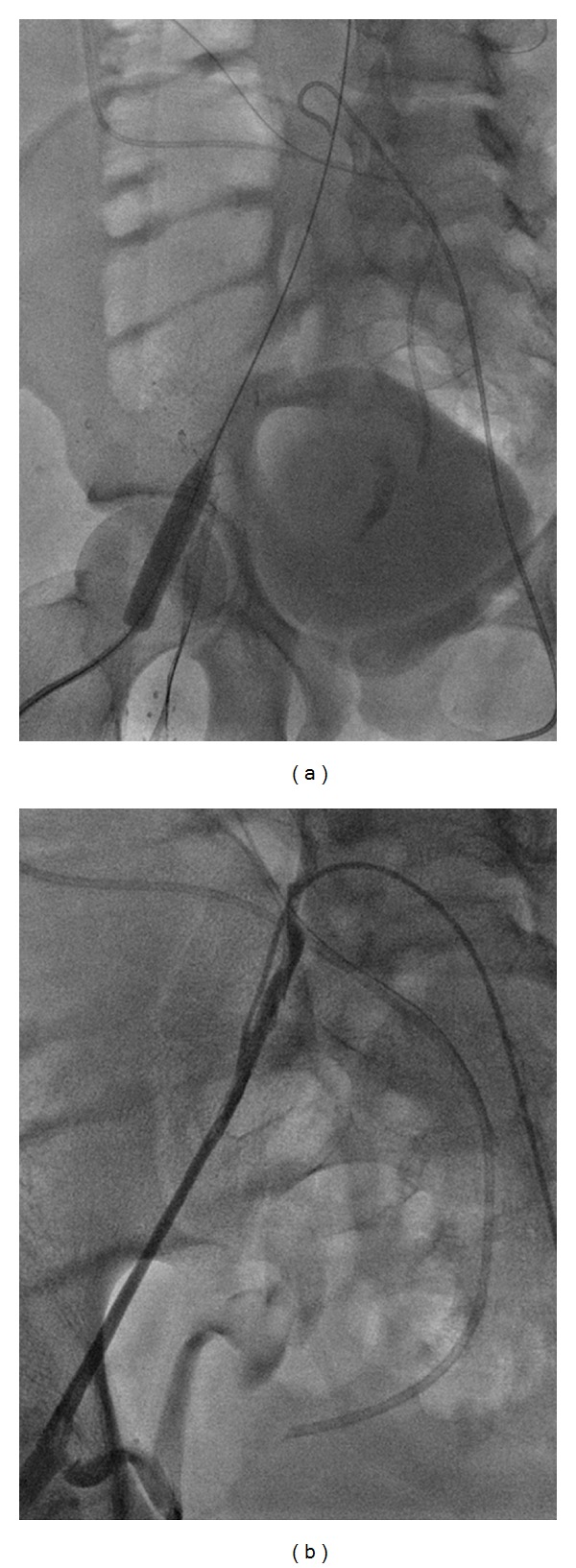
Balloon angioplasty of right iliofemoral system (a); follow-up venogram showing minimal improvement in diameter and flow via internal pelvic collaterals.

**Figure 5:**
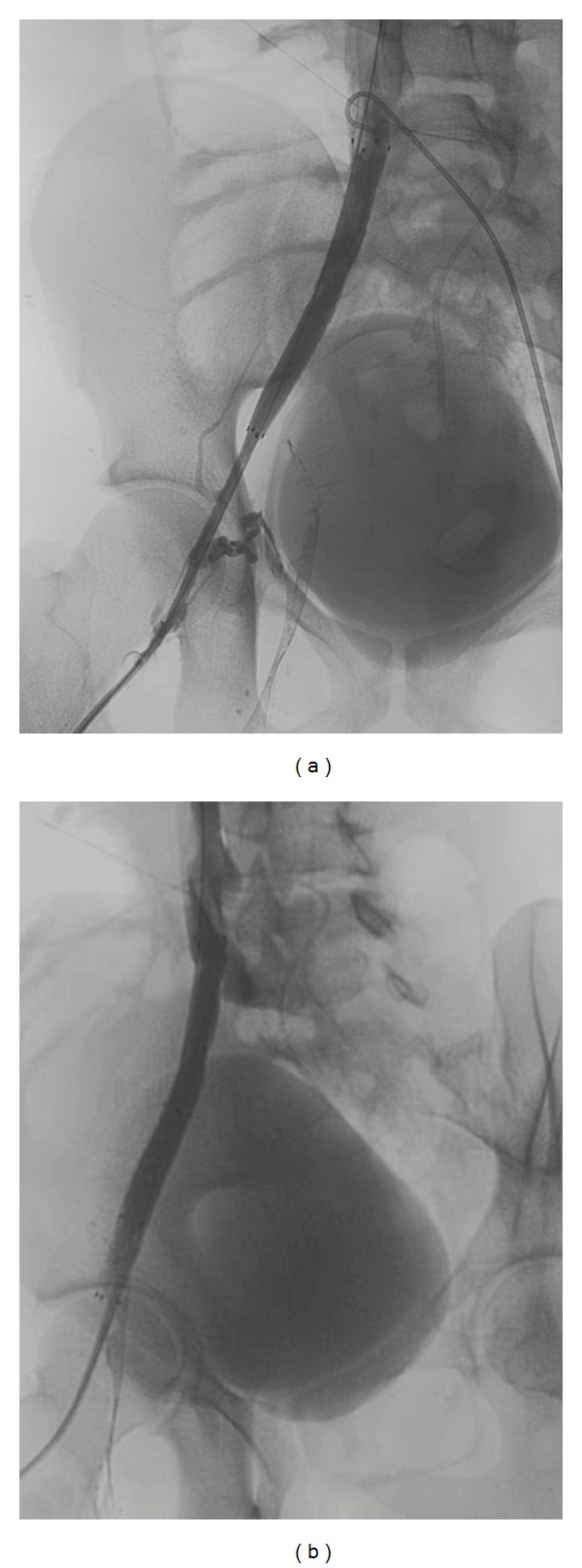
Follow-up venogram showing narrowed external iliac vein and collateral flow to internal iliac collateral (a); completion venogram (b) showing absence of flow in collateral veins as patent right iliac system.

After a detailed discussion with the patient and his parents, informed consent was obtained. Under moderate sedation, the right internal jugular vein (RIJ) was accessed and an attempt was made to cross the right common and external iliac lesions. This could not be accomplished. Therefore, a separate access was obtained under ultrasound guidance to the right and left common femoral veins below the varicosities. The guide wire from the RIJ to inferior vena cava (IVC) was captured. Through and through wire access was obtained from the neck to the right femoral area. The right iliac lesions were progressively dilated as demonstrated in Figure 4.  Figure 4(a) demonstrated the right iliac system during initial balloon angioplasty. Follow-up venogram in Figure 4(b) after initial balloon angioplasty showed little improvement. Flow is seen in pelvic collaterals to the internal iliac system. A diagnostic catheter was placed from the left common femoral to the IVC bifurcation and the location of the left common iliac vein was marked before stent placement. The results of 12 mm common and external iliac stent placements are shown in Figure 5. In Figure 5(a) some enlarged collateral veins still flowed into the right internal iliac vein. In Figure 5(b), completion venogram, the stent is extended well into the right external iliac vein; the pelvic collateral veins are not seen as venous flow is diverted into the right iliac system.

## 4. Discussion

It is estimated that over 30% of women will have chronic pelvic pain during life [1]. This chronic pelvic pain is often a manifestation of venous insufficiency of the gonadal and pelvic veins. In women, this is manifested by swelling in/of the vulva or vagina, as well as vulvar, buttock, and leg varicosities. Dyspareunia and abnormal menstrual bleeding might also result. Men are also affected; varicoceles are more common on the left system than on the right because of the origin of the left gonadal vein from the left renal vein. Interestingly, these findings were neither present nor associated with varicosities in this case. A more common cause of superficial pelvic varicosities is from reflux in the pelvic veins from venous insufficiency or from the so-called nutcracker syndrome caused by the compression of the left renal vein by the SMA and Aorta [2]. In addition, renal vein anomalies such as a retro aortic left renal vein might also be a cause of pelvic congestion syndrome [3]. Reyes et al. advocated the use of percutaneous embolotherapy in the treatment of adolescents with varicoceles [4]. This patient had an unusual presentation of chronic right iliac vein occlusion with these large varices acting as a collateral pathways redistributing the blood flow from the occluded vessels to the patent contra lateral iliac system. Obstruction rather than venous insufficiency was effectively diagnosed after comparing the MRI and venograpyh. In addition, compression of the renal vein between the SMA and aorta is another cause of left-sided gonadal venous hypertension and varicosities. This patient's presentation was different in that he had no significant scrotal varices, and these large varicosities were distributed from the right iliac fossa to the left as illustrated. The long-standing obstruction perhaps resulted from multiple femoral venous accesses as a 26-week premature infant. Moreover he did not have other clinical features of the Klippel-Trenaunay [5] or Weber syndrome [6] apart from these unusually located varicosities. Genetic testing was not considered. 

Normally, the skin and superficial fascia of the penis/clitoris and pubic region drain into the superficial external pudendal vein as well as the deep external pudendal veins. These veins might connect to the femoral vein or the greater saphenous veins. The right external iliac vein was significantly smaller than the left on MR venography as well as on intraprocedure venography. This vessel had to be mechanically dilated and treated with balloon angioplasty and stenting. While at first it might appear that these superficial varicosities were undesirable nuisances that were badly in need of sclerosing or excision, it was essential to fully evaluate not only the varicosities but also the feeding and outflow vessels. This evaluation helped us to conclude that the right external and common iliac veins were chronically blocked and these large varicosities provided an essential collateral pathway from the right lower extremity. Furthermore, longstanding subclinical occlusion, stenosis, or hypoplasia of the right iliofemoral system was probably worsened by recent onset of vigorous training for college sports. Since patient has done well in followup and the iliac fossa and suprapubic varicosities have remained decompressed and asymptomatic no further evaluation or treatment was deemed necessary.
